# Rapid response to the alpha-1 adrenergic agent phenylephrine in the perioperative period is impacted by genomics and ancestry

**DOI:** 10.1038/s41397-020-00194-5

**Published:** 2020-11-10

**Authors:** Stephane Wenric, Janina M. Jeff, Thomas Joseph, Muh-Ching Yee, Gillian M. Belbin, Aniwaa Owusu Obeng, Stephen B. Ellis, Erwin P. Bottinger, Omri Gottesman, Matthew A. Levin, Eimear E. Kenny

**Affiliations:** 1 Institute for Genomic Health, Icahn School of Medicine at Mount Sinai, New York, NY USA; 2 Department of Anesthesiology and Critical Care, Perelman School of Medicine, University of Pennsylvania, Philadelphia, PA USA; 3 Stanford Functional Genomics Facility, Stanford, CA USA; 4 Department of Medicine, Icahn School of Medicine at Mount Sinai, New York, NY USA; 5 The Charles Bronfman Institute for Personalized Medicine, Icahn School of Medicine at Mount Sinai, New York, NY USA; 6 Pharmacy Department, The Mount Sinai Hospital, New York, NY USA; 7 Department of Genetics and Genomics, Icahn School of Medicine at Mount Sinai, New York, NY USA; 8 The Hasso Plattner Institute for Digital Health at Mount Sinai, Icahn School of Medicine at Mount Sinai, New York, NY USA; 9 Department of Anesthesiology, Perioperative and Pain Medicine, Icahn School of Medicine at Mount Sinai, New York, NY USA; 10 Present Address: Invitae Corporation, San Francisco, CA USA

**Keywords:** Translational research, Genetic association study, Pharmacogenomics, Personalized medicine, Therapeutics

## Abstract

The emergence of genomic data in biobanks and health systems offers new ways to derive medically important phenotypes, including acute phenotypes occurring during inpatient clinical care. Here we study the genetic underpinnings of the rapid response to phenylephrine, an α1-adrenergic receptor agonist commonly used to treat hypotension during anesthesia and surgery. We quantified this response by extracting blood pressure (BP) measurements 5 min before and after the administration of phenylephrine. Based on this derived phenotype, we show that systematic differences exist between self-reported ancestry groups: European-Americans (EA; *n* = 1387) have a significantly higher systolic response to phenylephrine than African-Americans (AA; *n* = 1217) and Hispanic/Latinos (HA; *n* = 1713) (31.3% increase, *p* value < 6e−08 and 22.9% increase, *p* value < 5e−05 respectively), after adjusting for genetic ancestry, demographics, and relevant clinical covariates. We performed a genome-wide association study to investigate genetic factors underlying individual differences in this derived phenotype. We discovered genome-wide significant association signals in loci and genes previously associated with BP measured in ambulatory settings, and a general enrichment of association in these genes. Finally, we discovered two low frequency variants, present at ~1% in EAs and AAs, respectively, where patients carrying one copy of these variants show no phenylephrine response. This work demonstrates our ability to derive a quantitative phenotype suited for comparative statistics and genome-wide association studies from dense clinical and physiological measures captured for managing patients during surgery. We identify genetic variants underlying non response to phenylephrine, with implications for preemptive pharmacogenomic screening to improve safety during surgery.

## Introduction

Perioperative phenotypes, such as rapid response to drugs administered during surgery, constitute an integral part of medical practice, as the variation in drug response across individuals is a fundamental variable to consider before, during, and after surgery. One such perioperative phenotype is rapid physiologic responsiveness to short acting adrenergic agents administered to treat hypotension during surgery. Prolonged or recurrent episodes of hypotension during anesthesia and surgery are believed to be associated with increased morbidity and mortality [1–7], thus the use of these adrenergic agents is common. In the case of response to adrenergic agents, although anecdotal evidence has led clinicians to be aware of individual variation in response between patients, the lack of a rigorous replicable and quantitative phenotype prevents population-level comparisons, which may be linked to both genetic and environmental factors.

The past decades have seen tremendous growth in the use of clinical data from the Electronic Health Record (EHR) for the secondary purpose of research [8]. The establishment of EHR-linked biobanks of genomic data provide unprecedented opportunities for the types of translational and implementation research that drive precision medicine [9–11]. Much of biobank research has focused on longitudinal data captured at low temporal resolution (i.e., over months or years); for example, chronic disease conditions recorded in the EHR, or BP taken during routine annual outpatient office visits [12, 13]. Thus, most phenotypes observed in this manner are best suited for genomic discovery in the context of chronic (i.e., long-term) medical conditions. In contrast, pharmacogenomics has traditionally focused on the acute response to drugs administered in a controlled environment. There has been limited biobank-based research to date on the influence that genetics may have on the physiologic response to more acute clinical conditions, such as those experienced during surgery or during a hospital admission.

In this study, we focused our investigation on acute response to phenylephrine during the perioperative period. Phenylephrine, a selective α1-adrenergic receptor agonist, is one of the most commonly used agents for the treatment of intraoperative hypotension, with a very rapid onset when given intravenously and a short half-life of 15–20 min. Prior work focused on candidate genes had linked genetic variants in alpha-adrenergic receptors to differential phenylephrine response [14–18]. In addition, polymorphisms in *NOS3* [19] and *ACE* [20, 21] have been linked to heightened vascular responsiveness to phenylephrine. We had previously developed a rich database of high temporal resolution perioperative data extracted from the Mount Sinai Health System (MSHS) [22]. We used this database to generate robust phenotypic profiles of acute phenylephrine response during the perioperative period, and linked these phenotypes to genomic data from the multiethnic Mount Sinai Bio*Me* biobank. We explored whether genetic factors could help explain both the individual variability as well as the variation in response seen between individuals of differing ancestry.

## Materials and methods

### Study population

The Bio*Me* biobank is an EHR-linked biobank of over 60,000 participants, with ongoing enrollment since 2007, from the MSHS in New York City. Participants included in this analysis were recruited between 2007 and 2015, and consent to provide DNA and plasma samples linked to their de-identified EHRs. Participants provide additional information on self-reported ancestry through questionnaires administered upon enrollment. This study was approved by the Icahn School of Medicine at Mount Sinai’s Institutional Review Board. The study population consisted of *n* = 30,223 consented Bio*Me* participants aged 18 years or older (upon enrollment).

### Genomic data generation and QC

All patients were genotyped on the Illumina Global Screening Array through a collaboration with Regeneron Genetics Center. Genotype quality control steps were performed for all Bio*Me* participants and all genotyped variants as described in Belbin et al. [23]. Imputation was performed using the IMPUTE2 software and the 1000 genomes phase 3 reference panel [24, 25]. After QC filtering, a total of 42,246,687 SNPs were available for analysis.

### Linking perioperative and clinical data with genomic data

The Department of Anesthesiology maintains a linked database called iGAS that combines procedure-level data, including intraoperative events, duration of surgery, physiologic data, and medication administration data, linked to individual-level data in the Bio*Me* biobank, such as questionnaire, clinical, and genomic data. The architecture and content of iGAS have been previously described [22]. The resulting linked database is a rich resource of high resolution, high quality perioperative data that can be used to generate robust phenotype profiles. Further data parsing included categorization based on ICD-9 procedure codes, using the Healthcare Cost and Utilization Project Clinical Classification Software (HCUP CCS, REF https://hcup-us.ahrq.gov/toolssoftware/ccs_svcsproc/ccssvcproc.jsp) to map individual procedures to high level procedure categories (e.g., Digestive System). ICD-9 codes were used as the majority of data were captured prior to October 2015, and further our health care system did not transition to ICD-10 until late 2016. The Charlson comorbidity index (CCI) was calculated for each patient using administrative ICD-9-CM discharge diagnosis codes and an ICD-9-CM to comorbidity map with revised diagnosis weights [26, 27]. Calculation of the CCI was done using the R package *medicalrisk* [28]. The initial cohort consisted of 19,685 Bio*Me* participants linked to 55,104 procedures in iGAS. We filtered to include only patients of self-reported African American (AA, *n* = 4576), European American (EA, *n* = 5217), or Hispanic/Latino (HA, *n* = 6805) ancestries for downstream analysis.

### Phenotypic modeling

**Fig. 1:**
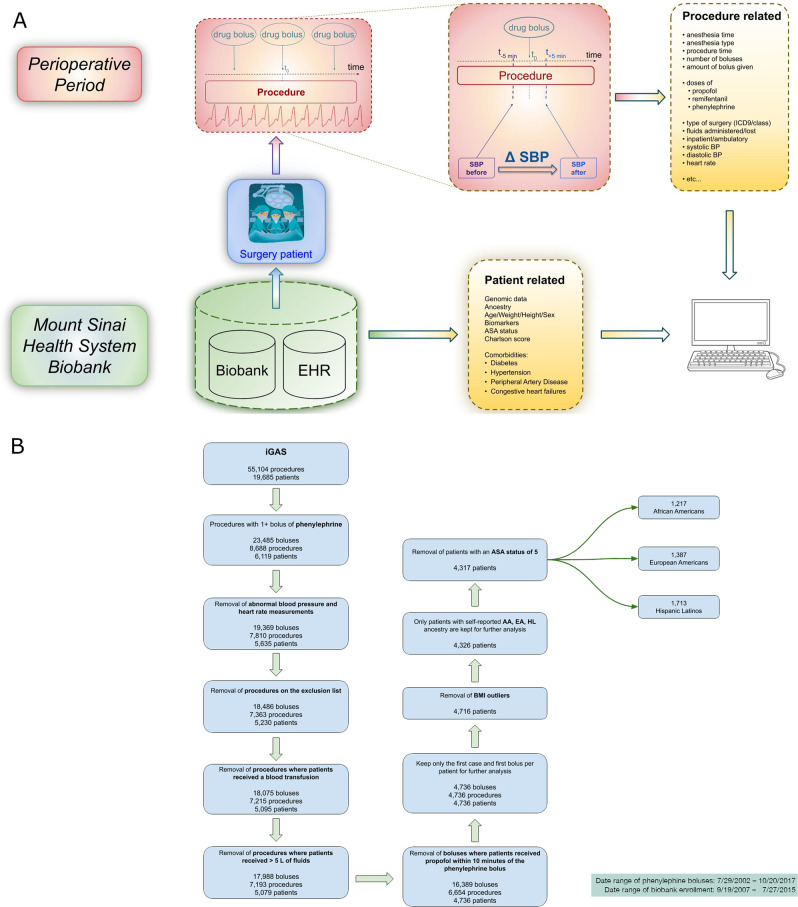
Data capture schema & Inclusion/Exclusion criteria. **A** Data capture schema. The raw phenotype is captured during surgery within a 10 min interval. Additional procedure-related information, as well as general patient-related variables, including genomic data, are combined through the analysis pipeline. **B** Inclusion/exclusion criteria. From an initial cohort of 19,685 iGAS patients matched to 55,104 procedures, the sequential exclusion criteria and filtering steps reduced the number of patients included in the study to 4317 comprising 1217 African Americans, 1387 European Americans, and 1713 Hispanic/Latinos.

Intraoperative blood pressure (BP) is typically measured either with a noninvasive blood pressure (NIBP) cuff at a frequency of once every 1–5 min, or continuously via invasive arterial line. Arterial line data are recorded as often as once every 15 s. BP measurements outside of normal physiological ranges (30–120 mmHg for diastolic BP, 30–130 mmHg for mean arterial pressure, 60–240 mmHg for systolic BP), as determined by validity flags in the electronic anesthesia record, were excluded as they likely represented measurement artifacts. To derive a phenotype suitable for further analysis, we defined a rapid drug response as the *difference* in the recorded *minimum* BP (systolic—SBP, diastolic—DBP, and mean—MAP) within a 5 min period *before* and the *maximum* BP in the 5 min period *after* a bolus of phenylephrine (Fig. 1A). Five-minute *before-* and *after-*windows were used to account for the fact that NIBP measurements and recordings are intermittent, as described above, and also that charting of bolus drug administration is often non-contemporaneous. For patients with an arterial line, this meant a measurement within as soon as 15 s before or after the drug dose was used. For patients with an NIBP, the closest reading could be as soon as within 1 min, if the NIBP cycle time was set to its shortest value. In either case (NIBP or invasive pressure monitoring), the longest delay between a bolus dose and a BP reading would be a maximum of 5 min. This ensured that the time relationship between drug administration and BP measurement was reasonably constant across all patients (Fig. 1). The final phenotypic measurement was thus the Δ SBP, DBP, and MAP response to a bolus of phenylephrine, within 5 min of administration.

### Case inclusion/exclusion criteria

Initial inclusion criteria were all Bio*Me* patients who had undergone anesthesia and received at least one bolus of phenylephrine intraoperatively. A diagram showing all exclusion steps is shown in Fig. 1B. Canceled cases or manually charted cases that had no automated recording of physiologic data were excluded. We also excluded all procedures of short duration and rapid turnover such as colonoscopies and endoscopies where drug bolus administration is known to often be significantly undercharted [29, 30]. Emergency procedures (~1%) were excluded, for several reasons: (1) these cases often involve blood loss and fluid shifts that may have profound effect on intravascular volume and responsiveness to phenylephrine, (2) emergency cases often receive blood products and fluid that are recorded with poor temporal resolution, and (3) charting of drug administration is often compromised and inaccurate during emergency situations. We also excluded procedures in which the patient received any blood transfusion, as blood is a potent vasoactive agent and intravascular volume expander. Additionally, we confirmed the statistical significance of the phenotypic difference between patients having received a blood transfusion and patients who did not receive a transfusion. We computed the correlation between the total amount of crystalloid administered and phenylephrine drug response, and excluded procedures with >5 L of fluid administration charted (~2%).

We performed a preliminary analysis to look for association between the intravenous anesthetic agent propofol and response to phenylephrine. Propofol is known to cause a predictable and often significant decrease in BP and has a similar onset (immediate) and duration (10 min) to that of phenylephrine. Based on the results of this preliminary analysis, we excluded phenylephrine bolus events within 10 min of a bolus dose of propofol. If that phenylephrine bolus was the only bolus the patient received during their procedure, the entire procedure was excluded. For patients with more than one anesthetic/procedure in the data set, only data from the first anesthetic was used. In order to be able to study the interindividual variability and to investigate the genetic basis of the phenotype, only one bolus-response was kept per patient. We kept the Δ SBP, DBP, and MAP associated with the first case and first bolus per patient.

Finally, we excluded BMI outliers (BMI > 100, likely due to data entry error) and procedures on critically ill patients with an American Society of Anesthesiologists (ASA) Physical Status of 5 (not expected to survive without surgery, *n* = 9). Such patients usually present with severe organ system derangement and little to no sympathetic reserve. After all patients, procedures, and bolus-response exclusion steps, our cohort contained a total of 4317 patients (1217 AA, 1387 EA, and 1713 HA), among which 3699 were genotyped and passed QC metrics (1082 AA, 1112 EA, and 1505 HA).

### Statistical analysis

Univariate analysis was used to select covariates to include in the genome-wide association study, with the level of significance set at *p* value < 0.05. To account for population-level differences, a genetic relationship matrix was calculated and the first ten principal components were included as covariates in the model [31, 32]. The final list of covariates used in the model for the association analysis were age through the use of age-adjusted *z*-scores, sex, BMI, ASA status, depth of anesthesia, phenylephrine bolus amount, total volume of fluid administered, and the first ten principal components. A SNP genotype association test was performed for all >47 million SNPs either imputed or genotyped directly, with the use of the GENESIS software package: a mixed model with polygenic random effects specified by a genetic relationship matrix was fitted using the *fitNullMM* function, and said model was then used to perform association tests with the *assocTestMM* function [31].

## Results

### Validation of phenotype modeling

After all exclusion steps, our clinical cohort contained a total of 4317 patients (1217 AA, 1387 EA, and 1713 HA). Demographics for the clinical cohort are shown in Table 1. Three thousand, six hundred ninety-nine had genotype data available and passed QC metrics (1082 AA, 1112 EA, and 1505 HA) (Fig. 1B). The average values over the entire cohort for the three different BP response measures were Δ SBP = 17 mmHg (±25), Δ MAP = 14 mmHg (±18), and Δ DBP = 11 mmHg (±14). To validate our method of extraction, we compared the observed drug response in our cohort with published estimates from Schwinn et al. (Schwinn and Reves [18]). Schwinn performed a small clinical trial on phenylephrine drug response in Europeans and reported a Δ MAP of ~15 mmHg after 100 μg bolus of phenylephrine. Our results were similar with an observed Δ MAP of 15.8 mmHg in European Americans. Likewise, for the entire cohort (all ancestries) we observe an average Δ MAP of 13.9 mmHg (Table 2).

**Table 1:** Demographic characteristics of the cohort used in the study.

Characteristic or outcome	Total	Self-reported ancestry			Differences between the three groups
		European-Americans	African-Americans	Hispanic/Latinos	
*n*	4317	1387	1217	1713	
Age (SD)	56.8 (±14.91)	61 (±14.28)	54.8 (±14.07)	54.9 (±15.31)	***
Sex (% female)	59.65%	44.99%	65.65%	67.25%	N.S.
BMI (SD)	29.7 (±8.13)	27.9 (±7.14)	31 (±8.9)	30.3 (±8.05)	***
ASA status					N.S.
1	101	26	27	48	
2	1378	4560	341	577	
3	2243	694	677	872	
4	595	207	172	216	
Charlson scores					**
0	1080	383	284	413	
1	306	95	84	127	
2	525	173	140	212	
3	309	88	90	131	
4	228	65	64	99	
5	151	47	58	66	
6	189	78	52	59	
7	104	19	40	45	
8	90	29	28	33	
9	40	6	19	15	
10	18	5	8	5	
11	15	1	9	5	
12	7	3	2	2	
13	2	0	1	1	
15	3	0	1	2	
18	1	0	1	0	
Hypertension (%)	1135 (26.29%)	374 (26.96%)	323 (26.54%)	438 (25.57%)	N.S.
Peripheral artery disease (%)	92 (2.13%)	34 (2.45%)	24 (1.97%)	34 (1.98%)	N.S.
Diabetes (%)	723 (16.75%)	146 (10.53%)	230 (18.90%)	347 (20.26%)	***
Congestive heart failure (%)	421 (9.75%)	121 (8.72%)	140 (11.50%)	160 (9.34%)	*
Major diagnostic category, *n* (% total)					N.S.
Undefined	291 (6.74%)	96 (6.92%)	85 (6.98%)	110 (6.42%)	
Infectious and parasitic diseases	45 (1.04%)	9 (0.65%)	16 (1.31%)	20 (1.17%)	
Neoplasms	609 (14.11%)	215 (15.5%)	171 (14.05%)	223 (13.02%)	
Endocrine; nutritional; metabolic; immunity	161 (3.73%)	30 (2.16%)	63 (5.18%)	68 (3.97%)	
Blood and blood-forming organs	2 (0.05%)	1 (0.07%)	0 (0%)	1 (0.06%)	
Mental Illness	6 (0.14%)	3 (0.22%)	2 (0.16%)	1 (0.06%)	
Nervous system; sense organs	177 (4.10%)	48 (3.46%)	40 (3.29%)	89 (5.20%)	
Circulatory system	548 (12.69%)	275 (19.83%)	110 (9.04%)	163 (9.52%)	
Respiratory system	83 (1.92%)	27 (1.95%)	23 (1.89%)	33 (1.93%)	
Digestive system	520 (12.05%)	155 (11.18%)	139 (11.42%)	226 (13.19%)	
Genitourinary system	296 (6.86%)	68 (4.90%)	89 (7.31%)	139 (8.11%)	
Complications of pregnancy; childbirth	242 (5.61%)	49 (3.53%)	84 (6.9%)	109 (6.36%)	
Skin; subcutaneous tissue	27 (0.63%)	4 (0.29%)	11 (0.9%)	12 (0.70%)	
Musculoskeletal; connective tissue	326 (7.55%)	80 (5.77%)	114 (9.37%)	132 (7.71%)	
Congenital anomalies	27 (0.63%)	12 (0.87%)	4 (0.33%)	11 (0.64%)	
Originating in the perinatal period	1 (0.02%)	0 (0%)	0 (0%)	1 (0.06%)	
Injury and poisoning	296 (6.86%)	73 (5.26%)	91 (7.48%)	132 (7.71%)	
Symptoms; signs; Ill-defined conditions	53 (1.23%)	16 (1.15%)	18 (1.48%)	19 (1.11%)	
Residual codes	12 (0.28%)	3 (0.22%)	4 (0.33%)	5 (0.29%)	
Main procedure category, *n* (% total)					***
Undefined	1291 (29.91%)	475 (34.25%)	332 (27.28%)	484 (28.25%)	
Nervous system	128 (9.23%)	42 (3.03%)	35 (2.88%)	51 (2.98%)	
Endocrine system	128 (9.23%)	47 (3.39%)	36 (2.96%)	45 (2.63%)	
Eye	99 (7.14%)	13 (0.94%)	24 (1.97%)	62 (3.62%)	
Ear	10 (0.72%)	1 (0.07%)	2 (0.16%)	7 (0.41%)	
Nose; mouth; pharynx	52 (3.75%)	12 (0.87%)	20 (1.64%)	20 (1.17%)	
Respiratory system	113 (8.15%)	41 (2.96%)	31 (2.55%)	41 (2.39%)	
Cardiovascular system	491 (35.4%)	221 (15.93%)	114 (9.37%)	156 (9.11%)	
Hemic and lymphatic system	27 (1.95%)	9 (0.65%)	10 (0.82%)	8 (0.47%)	
Digestive system	681 (49.1%)	210 (15.14%)	182 (14.95%)	289 (16.87%)	
Urinary system	154 (11.1%)	48 (3.46%)	38 (3.12%)	68 (3.97%)	
Male genital organs	56 (4.04%)	9 (0.65%)	15 (1.23%)	32 (1.87%)	
Female genital organs	153 (11.03%)	29 (2.09%)	52 (4.27%)	72 (4.20%)	
Obstetrical procedures	217 (15.65%)	47 (3.39%)	73 (6.00%)	97 (5.66%)	
Musculoskeletal system	422 (30.43%)	100 (7.21%)	152 (12.49%)	170 (9.92%)	
Integumentary system	209 (15.07%)	58 (4.18%)	71 (5.83%)	80 (4.67%)	
Miscellaneous	86 (6.20%)	25 (1.80%)	30 (2.47%)	31 (1.81%)

**Table 2:** Intraoperative characteristics of the cohort used in the study.

Characteristic or outcome	Total	Self-reported ancestry			Differences between the three groups
		European-Americans	African-Americans	Hispanic/Latinos	
*n*	4317	1387	1217	1713	
Anesthesia type					N.S.
General	2796	917	779	1100	
Mac	928	270	266	392	
Regional	593	200	172	221	
Anesthesia time (min)	187.9 (±147.8)	205.0 (±143.5)	179.6 (±135.2)	179.9 (±158.3)	***
Procedure time (mins)	114.1 (±96)	134.9 (±107.4)	105.5 (±86.8)	103.9 (±90.0)	***
Number of boluses per case	4.4 (±4.08)	4.9 (±5.0)	4.1 (±3.2)	4.2 (±3.7)	***
Number of cases per patient	1.4 (±0.9)	1.4 (±0.9)	1.4 (±0.9)	1.4 (±0.8)	N.S.
Mean bolus amount (micrograms)	107.2 (±44.5)	106.05 (±46.4)	108.8 (±45.9)	107 (±41.8)	N.S.
Median bolus amount (micrograms)	100	100	100	100	
Cases with propofol infusion (%)	1032 (23.90%)	326 (23.50%)	302 (24.82%)	404 (23.58%)	N.S.
Cases with remifentanil infusion (%)	390 (9.03%)	132 (9.52%)	111 (9.12%)	147 (8.58%)	N.S.
Cases with phenylephrine infusion (%)	179 (4.15%)	82 (5.91%)	39 (3.2%)	58 (3.39%)	***
Propofol infusion rate (mcg/kg/min)	21 (± 43.1)	20 (±41.5)	22 (±44.2)	21 (±43.5)	N.S.
Remifentanil infusion rate (mcg/kg/min)	0.01 (±0.13)	0.01 (±0.07)	0.01 (±0.05)	0.01 (±0.19)	N.S.
Phenylephrine infusion rate (mcg/kg/min)	0.02 (±0.35)	0.03 (±0.54)	0.01 (±0.06)	0.02 (±0.26)	N.S.
Total crystalloids administered (ml)	1057 (±1031.9)	1151 (±1082.9)	1020 (±1017.4)	1009 (±994.7)	***
Δ Systolic arterial pressure, mmHg (SD)	17.2 (±24.8)	20.1 (±24.5)	15.31 (±24.88)	16.35 (±24.89)	***
Δ Mean arterial pressure, mmHg (SD)	13.9 (±17.7)	15.8 (±17.4)	12.8 (±18.0)	13.2 (±17.7)	***
Δ Diastolic arterial pressure, mmHg (SD)	10.7 (±14.4)	12.1 (±13.8)	10.8 (±14.9)	10.0 (±14.5)	***
Δ Systolic Mann–Whitney vs. EA *p* value			5.42e−08	4.32e−05	
Δ MAP Mann–Whitney vs. EA *p* value			3.52e−06	2.35e−04	
Δ Diastolic Mann–Whitney vs. EA *p* value			4.79e−05	3.14e–04	

### Phenotype covariates

We found the presence of blood transfusion to be highly associated with phenylephrine drug response (Supplementary Fig. 5, *p* value = 5.40e−10). The distribution of total crystalloid administered was skewed and found to be highly correlated with phenylephrine drug response (Supplementary Fig. 6, *p* value = 4.90e−06). Preliminary analysis demonstrated a large confounding effect of the intravenous anesthetic agent propofol on the phenylephrine signal (*p* value = 5.764e−05 for a linear regression between Δ SBP and propofol bolus time). There was a significant difference in Δ SBP between patients with a propofol bolus administered within 10 min of a phenylephrine bolus and patients with no propofol bolus or propofol administered within more than 10 min of phenylephrine (Mann–Whitney *p* value < 0.003). The distribution of total crystalloid administered remained correlated with phenylephrine drug response after exclusion of procedures with >5 L of fluid administered (Supplementary Fig. 6, *p* value = 4.90e−06). We observed a nominal association between BMI and drug response (*p* value = 0.03), even after exclusion of outliers likely due to data entry errors. Age and gender were normally distributed in iGAS. Age was associated with drug response (*p* value = 4.55e−14, Supplementary Fig. 7) so age-adjusted *z*-scores were used for further analysis. Sex was only nominally associated with drug response (*p* value = 0.07), thus no exclusions or adjustments were made. ASA status (*p* value = 3.58e−07), depth of general anesthesia as measured by the Minimal Alveolar Concentration (MAC, *p* value = 5.23e−10, Supplementary Fig. 8) were associated with drug response in the univariate analysis. CCI was not associated with drug response. We found that phenylephrine response was slightly different between cardiovascular, musculoskeletal, and gastrointestinal surgeries, but the association was not statistically significant (*p* value = 0.6125). The bolus amount of phenylephrine given was not associated with the phenotype; however given the distribution of bolus amounts (Supplementary Fig. 9), the likely non-linearity of the dose–response relationship, and the absence of per patient dose–response curves, we kept phenylephrine bolus amount as a covariate in the model.

### Ethnicity impacts BP measures of rapid response to phenylephrine

**Fig. 2:**
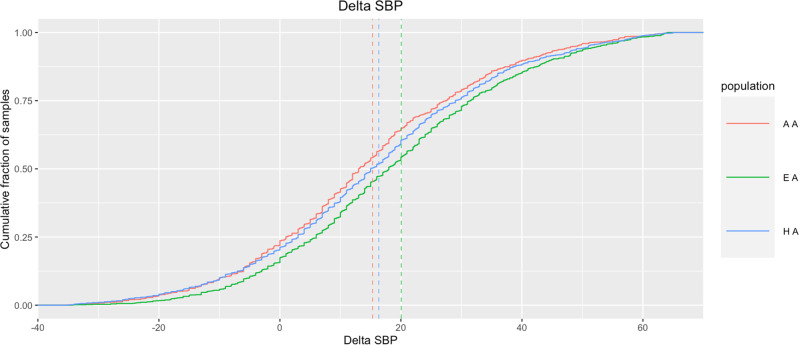
Drug response difference between populations. Cumulative distribution function for Δ Systolic Blood Pressure for African-Americans (in red), European Americans (in green), and Hispanic/Latinos (in blue). Average values for Δ SBP for AA, EA, HA are 15.31 mmHg (SE ± 0.71), 20.05 mmHg (SE ± 0.66), and 16.35 mmHg (SE ± 0.6) respectively.

In a population-stratified analysis, we observed that EA participants demonstrated a significantly heightened rapid response to phenylephrine compared to non-EA patients for all three measures (Table 2 and Fig. 2). The largest difference between populations was observed for Δ SBP (EA Δ SBP = 20 mmHg ± 24; HA Δ SBP = 16 mmHg ± 25; AA Δ SBP = 15 mmHg ± 25), thus this phenotype was used for all downstream analysis. After adjusting for age, sex, BMI, ASA status, phenylephrine bolus amount, depth of anesthesia, total volume of fluid administered, and accounting for self-reported (*via* validated survey) ancestry, significant differences remained for all three measures between EA participants and HA (Δ SBP, *p* < 0.032; Δ MAP, *p* < 0.021; Δ DBP, *p* < 0.008), and between EA and AA participants (Δ SBP, *p* < 5.13e−5; Δ MAP, *p* < 2.1e−4; Δ DBP, *p* < 3.3e−4).

### Genetic discovery

**Fig. 3:**
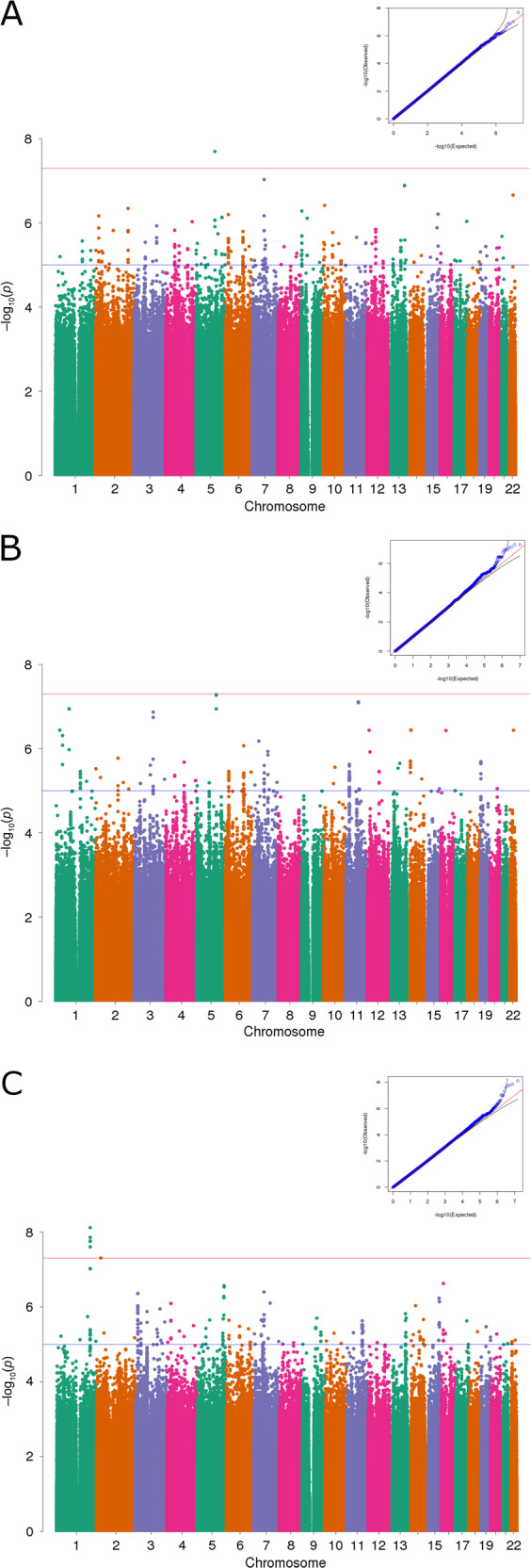
Manhattan plots and QQ plots of the genome-wide association studies. **A** GWAS performed on Δ SBP with the whole cohort. The red horizontal line denotes the genome-wide significance threshold for *p*-values (5e−8) while the blue horizontal line denotes a *p*-value threshold of 1e−5. The top SNP is rs145337816, located on chromosome 5, at locus 117211218 (in hg19 coordinates). The minor allele frequency is 0.46%, the imputation info score is 0.909. The association *p*-value is 2.02e−8. Lambda-gc = 1.0059. **B** GWAS performed on Δ SBP with European Americans. The top SNP is rs188427942, located on chromosome 5, at locus 122900413 (in hg19 coordinates). The minor allele frequency is 1.16 % in EA (0.57% in the whole cohort), the imputation info score is 0.759. The association *p*-value is 5.26e−8. Lambda-gc = 0.9976. **C** performed on Δ SBP with Hispanic/Latinos. The top SNP is rs6657443, located on chromosome 1, at locus 209189265 (in hg19 coordinates). The minor allele frequency is 7.63 % in HA (8.83% in the whole cohort), the imputation info score is 0.948. The association *p*-value is 7.62e−9. Lambda-gc = 1.0027.

**Fig. 4:**
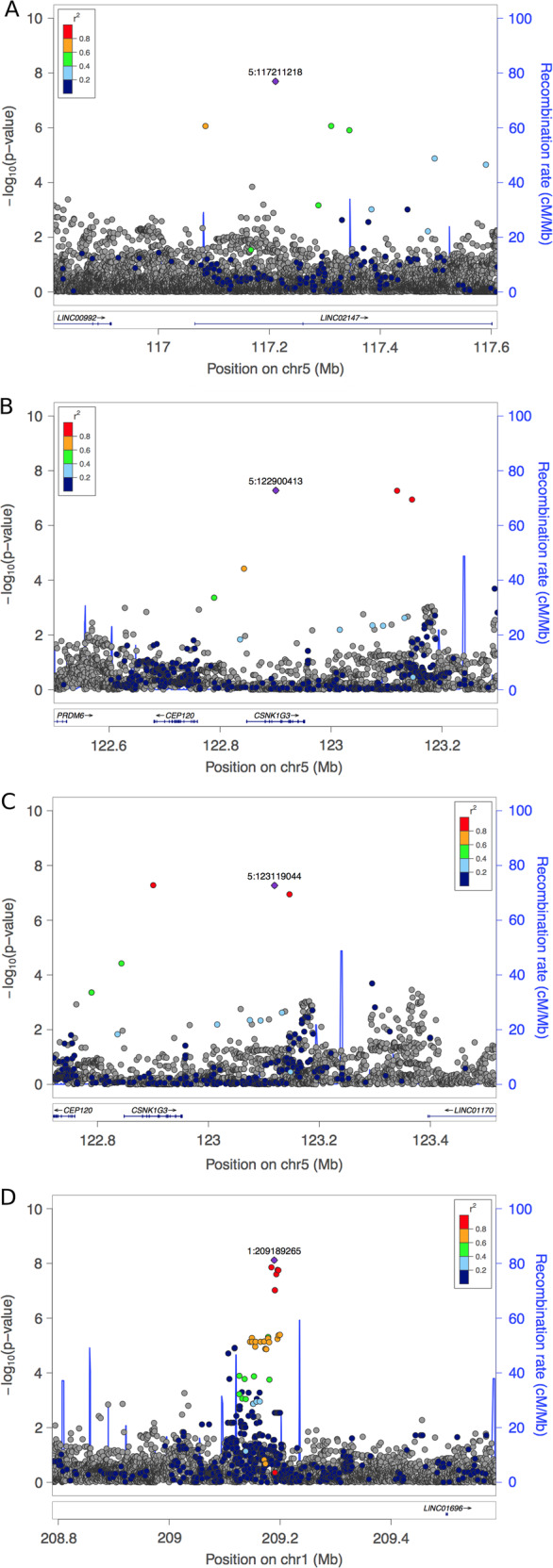
Locus Zoom plots showing patterns of linkage disequilibrium around top SNPs. **A** Patterns of LD around the top SNP rs145337816 for the GWAS performed on Δ SBP with the whole cohort. **B** Patterns of LD around the top SNP rs188427942 for the GWAS performed on Δ SBP with European Americans. **C** Patterns of LD around SNP rs147664194 for the GWAS performed on Δ SBP with European Americans. **D** Patterns of LD around the top SNPs for the GWAS performed on Δ SBP with Hispanic/Latinos.

We performed genome-wide association studies (GWAS) for systolic BP response to phenylephrine (Δ SBP), as well as Δ MAP and Δ DBP, across >47 million SNPs either imputed from 1000 genomes project or genotyped directly in 3699 individuals (1082 AA, 1505 HA, 1112 EA) altogether, as well as for each of the three populations separately. Across the three phenotypes (Δ SBP, Δ MAP, and Δ DBP) and the four population groupings (the whole cohort, AA, EA, and HA) we observed genome-wide significant associations (*p* value < 5e−8) in five different genome regions. Additionally, we observed two genome regions with suggestive associations at *p* values < 6e−8 and <7e−8 in EAs. Among these seven regions, five of them overlap variants previously associated with routine BP measurements (Table 3, Figs. 3, 4, Supplementary Figs. 1–3). The analysis performed on the HA population with the Δ SBP phenotype identified five adjacent genome-wide significant SNPs which are common in all three studied populations (all MAF > 7%), yet these variants are only associated with a significantly decreased systolic response in the HA population, thereby asserting the importance of ancestry as a factor influencing common alleles’ effect size in the case where these variants are causative. However, as the association is only present in the HA population, an alternative explanation is that the aforementioned SNPs are not causative and tag a variant only present in HA. Notably, these five SNPs are located at 1.27 Mb upstream of rs2761436, a variant previously associated with SBP in a study conducted in a population of mostly non-Hispanic Whites [13] (Table 3, Figs. 3, 4).

**Table 3:** Results of the genome-wide association studies performed with the three different phenotypes (Δ SBP, Δ MAP, and Δ DBP) and the four population groupings (the whole cohort, African Americans, European Americans, and Hispanic/Latinos).

Phenotype	Population	Chr	BP (hg19)	Beta	SE	*P* value	Associated gene(s)	rsid	Average carrier phenotype (mmHg)			MAF				Previous associations with BP phenotype^a^
									EA *n* = 1112	AA *n* = 1082	HA *n* = 1505	EA *n* = 1112	AA *n* = 1082	HA *n* = 1505	ALL *n* = 3699	
SBP	ALL	5	117,211,218	0.92	0.16	2.02E−08	LINC02147	rs145337816	37.3 ± 29.1	–	53.1 ± 36.2	0.56%	0.00%	0.33%	0.46%	
	EA	5	122,900,413	−1.10	0.20	5.26E−08	CSNK1G3	rs188427942	−6.8 ± 30.4	–	6.9 ± 42.3	1.16%	0.28%	0.34%	0.57%	rs1579036 (DBP), rs4530754 (PP), rs11750782 (SBP), rs373496042 (SBP), rs6891344 (DBP, SBP)
		5	123,119,044	−1.17	0.22	5.38E−08		rs147664194	−9.1 ± 31.6	–	–	1.01%	0.23%	0.27%	0.48%	
	HA	1	209,189,265	−0.39	0.07	7.62E−09	–	rs6657443	21.0 ± 28.0	14.0 ± 27.1	7.3 ± 24.0	7.87%	11.59%	7.63%	8.83%	rs2761436 (SBP)
		1	209,184,198	−0.36	0.06	1.39E−08		rs150793135	20.4 ± 27.6	14.2 ± 26.3	8.3 ± 23.7	7.80%	16.62%	9.03%	10.77%	
		1	209,195,248	−0.39	0.07	1.72E−08		rs6670957	21.0 ± 28.0	13.6 ± 26	7.2 ± 23.6	7.87%	10.29%	7.17%	8.28%	
		1	209,196,851	−0.40	0.07	1.78E−08		rs34954876	21.1 ± 28.2	13.6 ± 26	7.2 ± 23.7	7.76%	10.34%	7.14%	8.24%	
		1	209,193,053	−0.39	0.07	2.49E−08		rs139349180	21.0 ± 28.0	14.0 ± 26.5	7.4 ± 23.7	7.87%	10.40%	7.21%	8.32%	
MAP	ALL	13	30,950,119	0.14	0.02	1.97E−08	LINC01058, LINC00426, KATNAL1, HMGB1	rs145222507	16.6 ± 18.0	13.0 ± 17.4	15.3 ± 17.8	33.96%	39.10%	32.34%	34.82%	
		13	30,944,206	0.13	0.02	2.81E−08		rs1275187	16.0 ± 18.3	13.1 ± 17.5	15.1 ± 17.9	34.06%	39.72%	32.42%	35.05%	
		13	30,948,845	0.13	0.02	2.85E−08		rs1275192	16.0 ± 18.2	13.1 ± 17.3	15.3 ± 17.8	34.52%	39.81%	33.27%	35.50%	
		13	30,946,291	0.13	0.02	3.53E−08		rs1275189	16.0 ± 18.3	13.1 ± 17.6	15.1 ± 17.8	33.86%	39.64%	32.32%	34.92%	
		13	30,946,686	0.13	0.02	3.81E−08		rs1275191	16.0 ± 18.3	13.1 ± 17.6	15.1 ± 17.8	33.97%	39.64%	32.34%	34.97%	
		13	30,945,458	0.13	0.02	4.17E−08		rs2149850	16.0 ± 18.3	13.1 ± 17.5	15.1 ± 17.8	34.06%	39.72%	32.39%	34.94%	
	EA	1	41,519,758	0.49	0.09	6.38E−08	SCMH1, SLFNL1-AS1, SLFNL1, CTPS1	rs111908123	23.8 ± 19.2	13.0 ± 20.8	14.5 ± 18.8	5.90%	1.22%	4.25%	3.82%	rs11210029 (SBP)
DBP	AA	8	16,559,461	−1.27	0.22	1.13E−08	–	rs146535276	–	−8.1 ± 16.8	11.4 ± 19.4	0.05%	0.99%	0.33%	0.44%	rs11784910 (SBP, DBP), rs3793427 (AT), rs75902664 (DBP)
	AA	16	59,684,462	1.24	0.22	1.93E−08	APOOP5	rs143947120	–	27.5 ± 18.2	–	0.00%	0.99%	0.27%	0.40%	rs182346790 (PP), rs35300112 (SBP), rs1862746 (SBP)

The average carrier phenotypes are shown only for SNPs with ten carriers or more. SNPs previously associated with BP phenotypes in other studies are reported if they’re located within a 1 Mb genomic region overlapping the corresponding hit SNP, with the exception of rs2761436 (1.27 Mb).

^a^*PP* pulse pressure, *AT* arterial tonometry [42–45, 47–49, 55–58].

We performed a gene-based functional annotation analysis of the summary statistics of the GWAS using FUMA and MAGMA [33, 34]. Several genes share a high level of statistical significance across the gene-based analysis performed with the three different phenotypes, the three stratified populations and the joint population analysis. Six genes surpass a gene-based adjusted threshold for significance (Bonferroni adjusted *p* value < 1e−6) in all analyses: CNTNAP2, previously found to be associated with SBP and DBP in Mexican Americans [35]; MACROD2, associated with DBP in a multiethnic cohort [36]; CSMD1 and WWOX, associated with hypertension in Asian populations [37, 38]; DPP6, which contributes to transient outward current in Purkinje fibers [39]; SORCS2, associated with concentrations of IGFBP-3, a vasodilator [40, 41]. The complete results of the gene-based and gene set analysis are available in Supplementary Table 1.

We identified two subgroups in which the MAF was associated with an attenuated BP response: patients who either had no increase in BP in response to the bolus, or whose BP paradoxically declined after the bolus. The first group of non responders was identified through the analysis performed on the EA population, which identified two rare SNPs, rs188427942 (MAF EA: 1.16%) and rs147664194 (MAF EA: 1.01%) located in or near gene CSNK1G3 (Casein Kinase 1 Gamma 3), in regions previously associated with SBP, DBP, and PP (pulse pressure) [42–45]. CSNK1G3, which encodes a member of a family of serine/threonine protein kinases that phosphorylate caseins and other acidic proteins, is mostly expressed in fibroblasts [46] (Supplementary Fig. 4). On average, the 22 carriers of both SNPs have a Δ SBP of −9 mmHg (±32). In contrast the rest of the EA samples had an average Δ SBP of 20 mm Hg (±24). The second group of non responders was identified through the analysis performed on the AA population with the Δ DBP phenotype. Intergenic SNP rs146535276 (MAF AA: 0.99%) is located in a 1 Mb genomic region on chromosome 8 previously associated with SBP, DBP, and AT (arterial tonometry) [47–49]. On average, the 21 AA carriers of rs146535276 have a Δ SBP of −2 mmHg (±26) mmHg, while the rest of the AA cohort have a Δ SBP of 15 mmHg (±25) (Table 3, Figs. 3, 4, Supplementary Fig. 3). We also observed that carriers of rs145337816, a rare SNP only present in European Americans (MAF: 0.56%) and Hispanic/Latinos (MAF: 0.33%), constitute a small group of potential “super-responders”, i.e., patients with a significantly high response (Δ SBP_EA_ = 37 mmHg (±29) and Δ SBP_HA_ = 53 mmHg (±36)), with the caveat that results obtained on such a small number of individuals might not be reproducible.

We did not find genome-wide significant associations in *ACE* or *NOS3*, two genes harboring polymorphisms previously associated with response to phenylephrine, nor in adrenergic receptor genes. In particular, we report an absence of nominal association in our study of the *NOS3* polymorphism G894T (*p* = 0.19−1), previously linked to heightened vascular response to phenylephrine. Another variant previously associated with phenylephrine response, the insertion/deletion polymorphism rs1799752 in the *ACE* gene did not pass QC filters, and is thus not included in this analysis. Finally, we did not observe significant association at any of the adrenergic receptor genes (*ADRA1A, ADRA1B, ADRA1D, ADRA2A, ADRA2B, ADRA2C, ADRB1, ADRB2*, and *ADRB3*), and there was no significant enrichment in association at any gene, when compared with random regions of the genome of the same size. The results of the statistical tests for the most significant locus for *ACE*, *NOS3* and adrenergic receptor genes for each population and each phenotype tested are available in Supplementary Table 2.

### Systolic blood pressure genes association

**Fig. 5:**
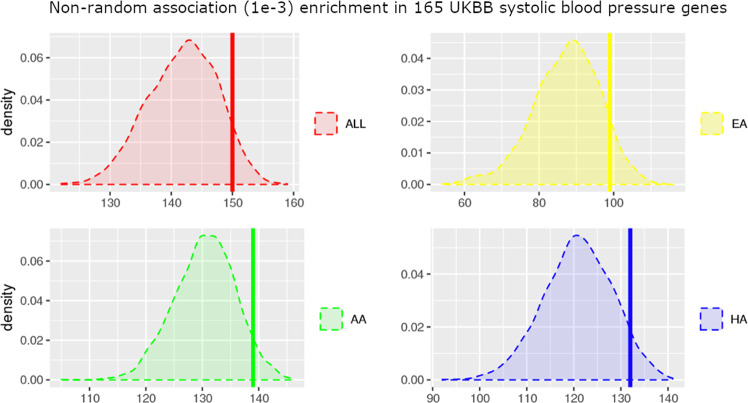
Association enrichment in systolic blood pressure genes. The four plots show the distribution of the number of SNP associations with a *p* value lower than 1e−3, when randomly selecting 165 genome regions (1000 permutations) for the four different population groups (the whole cohort in black, European Americans in green, African Americans in red, Hispanic/Latinos in blue). The vertical bars correspond to the number of genes out of the 165 genes identified in the UKBB cohort to be associated with systolic blood pressure which obtain a *p* value lower than 1e−3 when looking for association with Δ SBP in our four cohorts. The 165 systolic blood pressure genes are nominally enriched in association with Δ SBP in European Americans (*p* value = 0.096), Hispanic/Latinos (*p* value = 0.069), African Americans (*p* value = 0.060), and in the whole cohort (*p* value = 0.083).

We observed nonrandom enrichment in association (significance threshold set at *p* < 0.001) with systolic drug response in 165 loci previously reported to be associated with systolic BP in the UK BioBank cohort [50]. These 165 loci are centered around single nucleotide polymorphisms (SNPs) reported as being associated (*p* < 5e−8) with systolic BP in a genome-wide association study performed on 361,402 individuals of mostly European ancestry [50]. To explore whether the genetic loci underlying SBP as measured in routine clinical visits may impact the Δ SBP response to phenylephrine administration during surgery, we investigated whether these loci were enriched in the GWAS signals. First, we counted the number of times we observed a *p* value of association less than 10^−3^ in all 165 regions. Next, we counted the number of times we observed a *p* value of association <10^−3^ in 1000 random selections of 165 regions of similar genomic size, in order to generate a distribution of random signal across the genome (Fig. 5). Out of the 165 independent genome regions associated with SBP in the UK BioBank cohort, 150, 139, 99, and 132 regions were associated with Δ SBP in our complete cohort, and in AA, EA, and HA individuals respectively. Alternatively, when randomly selecting 165 regions of the genome, 141.9 (±5.69), 130.4 (±5.41), 87.4 (±8.95), and 120.9 (±7.34) regions are associated with Δ SBP in our complete cohort, and in AA, EA, and HA individuals respectively (1000 random selections were performed). Comparing these random distributions with the ratio of systolic BP regions associated with Δ SBP in the four cohorts allows us to establish *p* values of 0.096, 0.069, and 0.06 for EA, HA, and AA participants respectively and of 0.093 for the full cohort. These results, albeit not statistically significant, indicate a positive trend of potentially shared genetic architecture underlying both phenylephrine response and systolic BP.

## Discussion

In the present study, we successfully used extant clinical data recorded during surgery for the secondary purpose of exploring genomic factors underlying rapid response to phenylephrine, a therapeutic used to treat BP. We underscored the importance of defining a rigorous phenotype to represent the dose–response to phenylephrine during surgery. Reassuringly, the results of our derived phenotype closely matched the BP change observed by Schwinn and Reves 30 years ago [18]. This consistency validates both Schwinn’s original results obtained in a small cohort, as well as our contemporary results derived from routine clinical care. Our phenotypic modeling process allowed us to perform population-level comparisons, notably highlighting statistically significant differences across self-reported ancestries. We found that self-identified Europeans had a significantly heightened response to phenylephrine administered intraoperatively compared to other populations. We tested several hypotheses to explain this difference, including, age, gender, body mass index, level of anesthesia, concurrent medications, and co-morbidities (Table 1). None of the aforementioned variables explained the difference in phenylephrine response across populations. Differences in drug response between populations provide evidence that genetic ancestry and/or unmeasured environmental factors could account for the trait variability across populations, as is the case with other drug response or pharmacogenomics phenotypes [51, 52].

Using this derived trait in a GWAS allowed us to discover novel loci in the genome that could explain variability in drug response to phenylephrine. Notably, five out of the seven independent genomic regions harboring SNPs associated with the three derived phenotypes were previously associated with related BP phenotypes. In a gene-based functional annotation analysis of the GWAS results, we linked six genes in these regions to previously reported findings for SBP, DBP, and hypertension. We observed suggestive evidence to support the enrichment of genes previously associated with SBP. This evidence indicates a general shared genetic architecture between systolic drug response and SBP. Conversely, we did not find evidence of significant genetic association at any of the adrenergic receptor genes. While it had previously been observed in small cohorts of patients undergoing cardiac surgery with CPB that carriers of the G894T polymorphism of NOS3 have a significantly heightened vascular responsiveness to phenylephrine [19], we did not observe this effect in our cohort. We were not able to confirm a similar effect in carriers of the insertion/deletion polymorphism of ACE [20, 21], as this specific variant was not included in our analysis. Taken together, these findings suggest that, on average, the underlying etiology of BP has a larger effect than pharmacogenetic variation on an individual’s response to phenylephrine administered intravenously during surgery

However, our experimental design also allowed us to identify rare variants whose carriers are phenylephrine non responders. For example, there were 22 European American participants (2%) carrying 2 variants and 21 African American participants (2%) carrying a single variant in our study each associated with the absence of response to phenylephrine. Being able to predict non responders to a particular therapeutic prior to surgery based on a small number of variants has important clinical implications, especially in an era of personalized medicine, where the number of patients being genotyped or sequenced grows exponentially. Due to the novelty of the phenotype we developed in this study, we are not able to independently validate this finding in other cohorts, and future studies are needed to replicate this finding and evaluate medical utility. Finally, we observed 851 other non responders (23%) in our study who do not carry these variants, indicating that there could be other genetic and/or nongenetic factors at play.

Our study adds to the existing body of knowledge by significantly increasing the number of sampled individuals and assessing Δ MAP, Δ SBP, and Δ DBP in patients of diverse ancestries, as the extent to which African Americans and Hispanic/Latinos respond to intravenous phenylephrine administration was previously unknown. There are parallels between our study and a recent publication by Zhang et al. but with several important differences [53]. That study analyzed phenylephrine infusions rather than boluses, was limited to patients of European ancestry and did not identify genome-wide significant loci. Conversely, thanks to our study design and multiethnic cohort, we were able to highlight significant differences between the three populations for the derived phenotypes and detect population-specific genome-wide significant loci associated with phenotypes. These findings allow us to discriminate the roles of pharmacogenomics and vascular biology in a novel perioperative phenotype, and demonstrate that genetic determinants underlie individual-level response to phenylephrine administered during surgery.

### Limitations

Our approach entails some caveats and should be considered as exploratory science rather than hypothesis-based. Even at the scale of hospital-wide biobanks, the application of stringent sample filtering and removal steps, which are paramount when studying a phenotype measured in the “real-world”, dynamic environment of an operating room, prevents us from reaching sample sizes large enough to replicate this method on less commonly used drugs or to extrapolate a polygenic risk score. It is important to highlight that using only a single response does not allow us to build a dose–response curve for each patient and it is possible that if we had chosen a different bolus to analyze, we would have seen somewhat different results. However, the majority of patients were given the same dose, which is a standard dose used to treat hypotension in the operating room. Furthermore, while we did include depth of anesthesia at the time of the phenylephrine bolus as a covariate, the BP response observed was likely also dependent on the degree of surgical stimulation (or lack thereof) and volume status at the time of the bolus. These variables were not captured and remain as potential unmeasured residual confounders.

Thanks to the diversity of our biobank, our multiethnic study was balanced enough to allow us to observe significant differences in drug response across self-reported population groups. However, given the expanse of our sample set and the inherent statistical limitations of the methods used, genomic signals recorded should be reckoned as potential association indications requiring further downstream analysis rather than definitive assessments of the functional role of genes mentioned, particularly for low frequency variants which have a susceptibility of being false positives. Cardiac output, which is not routinely measured outside of cardiac surgery operating rooms, is a significant residual confounder of the BP response to any pressor. The incidence of congestive heart failure (CHF), which is usually a low cardiac output state, was significantly higher among African Americans in our cohort. It is possible that this is partially responsible for the blunted SBP response we observed in this group, however, a multivariate analysis showed no significant association between CHF and SBP in our cohort. We were able to identify groups of nonresponders carrying rare variants, however given the small number of patients carrying these variants, several potential confounders might be at play, and these results should be considered preliminary until confirmed by further prospective studies.

As biobanks and genomic databases are constantly increasing in size, limitations in sample size will gradually disappear. Moreover, federated datasets and data-sharing across multiple institutions are paving the way toward sample sizes orders of magnitude larger than currently available ones, leading to significant increases in statistical power and precision [8, 54]. Approaches like the one used in this paper, leveraging the union of genomics and extensive clinical data capture, as well as statistical tools borrowed from the field of artificial intelligence to customize treatment, will lead to tailored dosage and anesthetic plans, thereby constituting a true paradigm change in the practice of surgery and making it the next field where patients can expect to benefit from precision medicine.

## Supplementary information

Supplementary Materials

Supplementary Table 1

Supplementary Table 2

List of Supplemental Material
