# Supplementary material for: Rapid response to the alpha-1 adrenergic agent phenylephrine in the perioperative period is impacted by genomics and ancestry
Source: Pharmacogenomics J. 2020 Nov 10;21(2):174–89. doi: 10.1038/s41397-020-00194-5 (PMC7997806; doi:10.1038/s41397-020-00194-5)
Supplement: Supplementary file 4 — List of Supplemental Material [file 41397_2020_194_MOESM4_ESM.docx]

**List of Supplementary Materials**

**Supplementary Figure 1.** Manhattan plot and QQ-plot of the GWAS performed with the delta MAP phenotype and the full cohort. Locus zoom of the region containing 6 genome-wide significant SNPs (rs145222507, rs1275187, rs1275192, rs1275189, rs1275191, rs2149850).

**Supplementary Figure 2.** Manhattan plot and QQ-plot of the GWAS performed with the delta MAP phenotype and the European American cohort. Locus zoom of the region containing the top SNP (rs111908123).

**Supplementary Figure 3.** Manhattan plot and QQ-plot of the GWAS performed with the delta DBP phenotype and the African American cohort. Locus zoom plots of the two regions containing the two genome-wide significant SNPs (rs146535276, rs143947120).

**Supplementary Figure 4.** Tissue expression of CSNK1G3.

**Supplementary Figure 5.** Phenotype difference between patients having received blood transfusions during surgery and patients without transfusions.

**Supplementary Figure 6.** Association between the total amount of crystalloid administered and the phenotype.

**Supplementary Figure 7.** Association between age and the phenotype.

**Supplementary Figure 8.** Association between Minimal Alveolar Concentration and the phenotype.

**Supplementary Figure 9.** Distribution of bolus amounts across all patients and all procedures.

**Supplementary Table 1.** Results of the functional annotation.

**Supplementary Table 2.** Results of the statistical tests for the most significant locus for *ACE*, *NOS3* and adrenergic receptor genes.

**Regeneron Genetics Center Banner Author List and Contribution Statements**

**Charles Bronfman Institute for Personalized Medicine Genomics Group Banner Author List and Contribution Statements**
